# Impact of a pulmonary rehabilitation program on social disadvantage and physical activity data of postCOVID19 patients: A North-African pilot study

**DOI:** 10.12688/f1000research.126301.1

**Published:** 2022-10-27

**Authors:** Wafa BENZARTI, Emna TOULGUI, Amine GHRAM, Chiraz RAHMANI, Sana AISSA, Ines GHANNOUCHI, Imene GARGOURI, Amani SAYHI, Asma KNAZ, Walid OUANES, Sonia JEMNI, Helmi BEN SAAD

**Affiliations:** 1 Department of Pneumology, Farhat HACHED Hospital, Sousse, Tunisia; 2 Department of Physical Medicine and Rehabilitation,, Sahloul Hospital,, Sousse, Tunisia; 3 Research laboratory “Heart failure, LR12SP09”, Hospital Farhat HACHED of Sousse, Sousse, Tunisia; 4 Laboratory of Physiology, Faculty of Medicine of Sousse, University of Sousse, Sousse, Tunisia

**Keywords:** Handicap, Health Status, North Africa, Physical Rehabilitation, SARS-Cov-2

## Abstract

Background

In addition to the cardiorespiratory, muscular, and neurological manifestations, coronavirus disease 2019 (COVID-19) alters patients’ health-related quality of life (HRQoL), induces a large variety of psychiatric manifestations, and reduces mobility and motor activity. Several studies have raised the impact of a pulmonary rehabilitation program (PRP) on social disadvantage ( *e.g.,* HRQoL, anxiety, depression) and physical activity of COVID-19 patients, but very few have been performed in low-income countries. This study aimed to investigate the impact of a PRP on post-COVID-19 HRQoL, hospital anxiety and depression (HAD), and physical activity in Tunisian post-COVID19-patients.

Methods

This was a cross-sectional study in an outpatient care setting. Patients with post-COVID-19 were included. They completed an interview (including three questionnaires) before and after a PRP (three sessions/week for four weeks, each session was 70 minutes in duration, PRP items: aerobic cycle endurance, strength training, and education). The VQ11 questionnaire assessed functional dimension, psychological dimension, relational dimension, and total score; HAD appraised depression and anxiety; and Voorrips physical activity assessed daily activity, physical activity, leisure activity, and total scores. Data were expressed as mean±standard deviation in PRP change (PRP change=after-PRP values − before-PRP values).

Results

In total, 14 moderate to severe post-COVID-19 patients (61±4 years) were included. The PRP significantly improved the ***i)*** functional, psychological, and relational dimensions, and the VQ11 total score by 1.79±1.58 (p=0.0033), 2.00±2.15 (p=0.0108), 1.57±1.50 (p=0.0077), and 5.36±3.97 (p=0.0015), respectively; ***ii)*** HAD anxiety and depression scores by 2.07±2.40 (p=0.0076), and 2.57±3.08 (p=0.0058); and ***iii)*** physical activity and total scores by 1.75±2.44 (p=0.0251), and 1.78±2.65 (p=0.0341), respectively.

Conclusion

The PRP improved HRQoL, HAD, and physical activity of Tunisian post-COVID-19 patients.

## Abbreviations list

aPRP: after pulmonary rehabilitation program

BMI: body mass index

bPRP: before pulmonary rehabilitation program

EQ 5 D: Euroqol five domains

EQ-5D-5L: Euroqol five domains 5 levels

GAD-7: generalized anxiety disorder-7 questionnaire

HAD: hospital anxiety and depression

HR: heart rate

HRQoL: health-related quality of life

PRP: pulmonary rehabilitation program

RT-PCR: reverse transcriptase-polymerase chain reaction

SAS: self-rating anxiety scale

SD: standard deviation

SDS: self-rating depression scale

VAS: visual analogue scale

## Introduction

Coronavirus disease 2019 (COVID-19) is an infectious disease involving the respiratory system and several extra pulmonary organs. ^1^ COVID-19 has clinically diverse manifestation ranging from asymptomatic presentation to critical illness. ^2^ In people recovering from COVID-19, there is some concern regarding the potential long-term sequelae and associated impairment of functional capacity since clinical sequelae may persist after acute-COVID-19. ^3^ According to one guideline ( www.nice.org.uk/guidance/ng188 2020), signs and symptoms of COVID-19 from 4 to 12 weeks after the onset of the first symptoms are defined as “ongoing symptomatic COVID-19”, while COVID-19 sequelae that last >12 weeks are summarized by terms such as “post-COVID-19 syndrome”, “long-COVID-19”, or “post-acute sequelae of infection by severe acute respiratory syndrome coronavirus 2”. ^4^ The main manifestations reported by patients are organic such as chest pain, breathlessness, fatigue, and reduced mobility. ^3^

In post-COVID-19 patients, psychological manifestations are noted. ^5^^–^^8^ Indeed, COVID-19 was reported to alter health-related quality of life (HRQoL). ^5^^–^^9^ Three months after symptom onset, around 75% of hospitalized COVID-19 patients identify unusual patient-reported outcomes, with 33% of patients identifying at least moderate deficiencies in major dimensions of HRQoL. ^8^ The relationship between COVID-19 and altered HRQoL could be related to severity at admission, hypoxaemia, systemic inflammation, respiratory support during hospitalization, and lack of contact with family and loved ones during quarantine or hospitalization. ^10^^,^^11^ Persistent symptoms post-acute phase could explain the persistence of a poor HRQoL. ^12^ COVID-19 was reported to possibly lead to symptoms of depression and anxiety, and lack of motivation. ^5^^–^^9^ Among the described health problems of post-COVID-19, neuropsychological impairments are common (47%), with a high prevalence of psychological disorders such as augmented levels of stress, anxiety, and depression. ^13^^–^^16^ Since HRQoL is a multidimensional concept that includes domains related to physical, mental, emotional, and social functioning, it is important to focus on and improve it. ^17^

During the virus’s speedy spread in the absence of a COVID-19 vaccine, numerous states instigated restrictive procedures (e.g., stay-at-home instructions, closures of parks and fitness and recreation centers). ^18^ These procedures led to a rise in physical inactivity, which is recognized as a risk factor for COVID-19 severity and mortality. ^18^^,^^19^ COVID-19 patients have reduced mobility and motor activity, which leads to an increase in unhealthy lifestyle habits, raising the risk of diseases. ^20^ In patients with chronic conditions, one clinical guideline recommend to increase their physical activity and emotional participation in everyday activities. ^21^ Physical activity has been proven to be effective in improving both mental and physical health and continues to be the greatest method to preserve well-being of the body, to guarantee quotidian activities, and to preserve a good function of the cardiorespiratory and muscular chain. ^21^^,^^22^ Thus, as in chronic cardiorespiratory diseases, COVID-19 patients could benefit from a pulmonary rehabilitation program (PRP). ^23^^,^^24^

The European Respiratory Society taskforce recommends that COVID-19 patients with a need for rehabilitative interventions should receive a PRP. ^25^ Most publications carried out in high-income countries investigated the impact of PRP on submaximal exercise data on COVID-19 patients. ^26^^–^^37^ In COVID-19, a PRP is an excellent management axis, but studies related to its impact on patients’ social disadvantage or physical activity are scarce. ^24^^,^^26^^–^^29^^,^^31^^–^^34^ Four systematic reviews demonstrated the feasibility and efficiency of a PRP in the management of post-COVID-19 patients. ^35^^–^^38^ Almost all of the retained studies were performed in specialized rehabilitation units from high-income countries. In low-income countries, the potential benefit of a PRP after COVID-19 is unclear.

Since no previous study from a low-income country raised the impact of an ambulatory PRP on HRQoL, psychological manifestation, and physical activity of COVID-19 patients, the aim of this cross-sectional study was to examine the impact of a PRP on social disadvantage (HRQoL, anxiety and depression), and physical activity of Tunisian COVID-19 patients. PRP will be considered ‘efficient’ if the PRP change (ΔPRP=after (aPRP) minus before (bPRP) PRP values) for the main outcome of this study (HRQoL total score) is statistically significant.

## Methods

**Figure 1.**
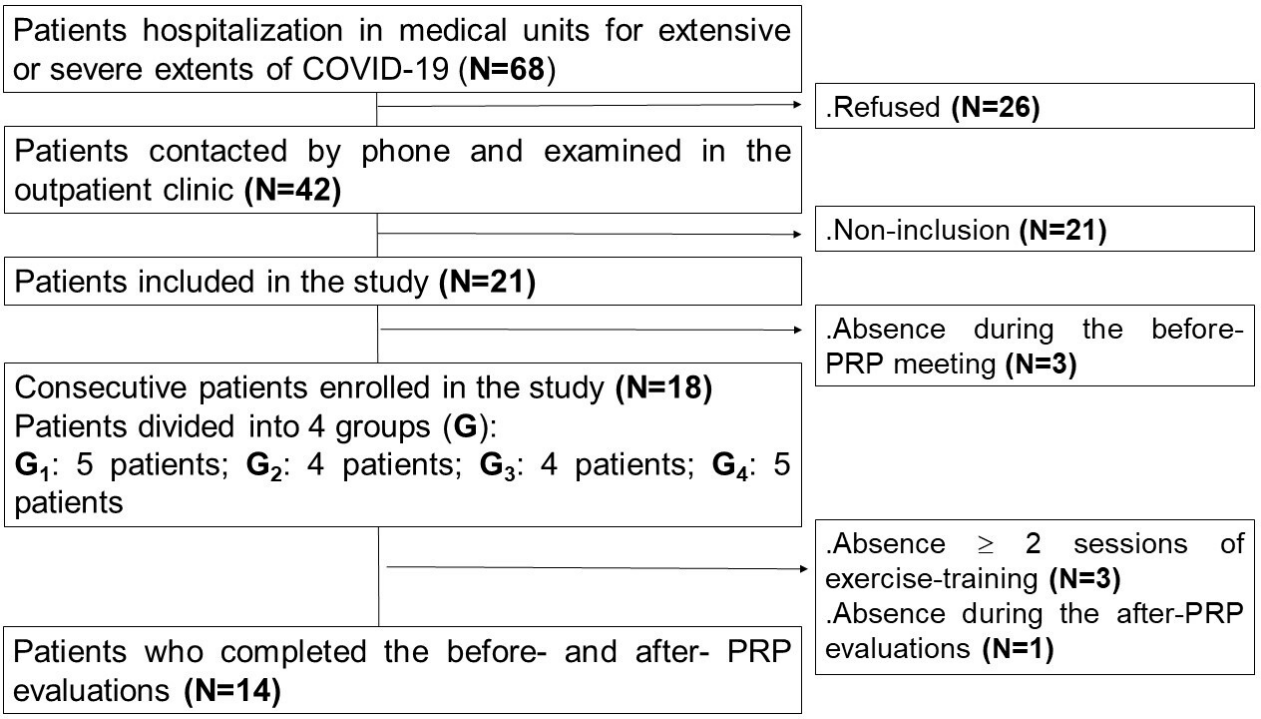
Study protocol. **COVID-19**: coronavirus disease 2019. **PRP**: pulmonary rehabilitation program.

The present study is part of a project including two parts. The first part aimed to evaluate the impact of a PRP on the 6-minute walk test performed by COVID-19 patients. ^24^ Meanwhile, this research constitutes the second part of this project. Figure 1 details the project flowchart.

### Study design

This was a cross-sectional study conducted from 2 ^nd^ February 2021 to 26 ^th^ September 2021 by a multidisciplinary team including the department of pulmonology and department of physiology and functional explorations at the Farhat HACHED hospital, Sousse, Tunisia, and the department of physical medicine and rehabilitation at the Sahloul hospital, Sousse, Tunisia. During each study step, all recommended preventive measures to fight against COVID-19 transmission were applied. The study was conducted following the guidelines established by the STROBE statement. ^39^

### Study population

The target population was COVID-19 patients hospitalized (during February to June 2021) in the COVID-19 units of the aforementioned pulmonology and physical medicine departments. Only male COVID-19 patients aged > 50 years, with a chest-computed tomography during the hospitalization period showing an extensive/severe extent of parenchymal lung injury ^40^ were included in this study. COVID-19 patients admitted into an intensive care unit were excluded. Absence during two or more exercise training sessions or the aPRP evaluation session was applied as an exclusion criterion.

COVID-19 diagnosis was confirmed by reverse transcriptase-polymerase chain reaction (RT-PCR). ^41^ All patients underwent chest-computed tomography. The following two classifications were applied: ***i)*** chest-computed tomography classification, which consisted of five levels based on the extent of parenchymal lung injury: absent or minimal (<10%), moderate (10–25%), extensive (25–50%), severe (50–75%), and critical (>75%), ^40^ and ***ii)*** clinical classification ( https://www.who.int/publications/i/item/WHO-2019-nCoV-clinical-2021-2), including four levels (mild, moderate, severe, and critical).

### Applied protocol

**Figure 2.**
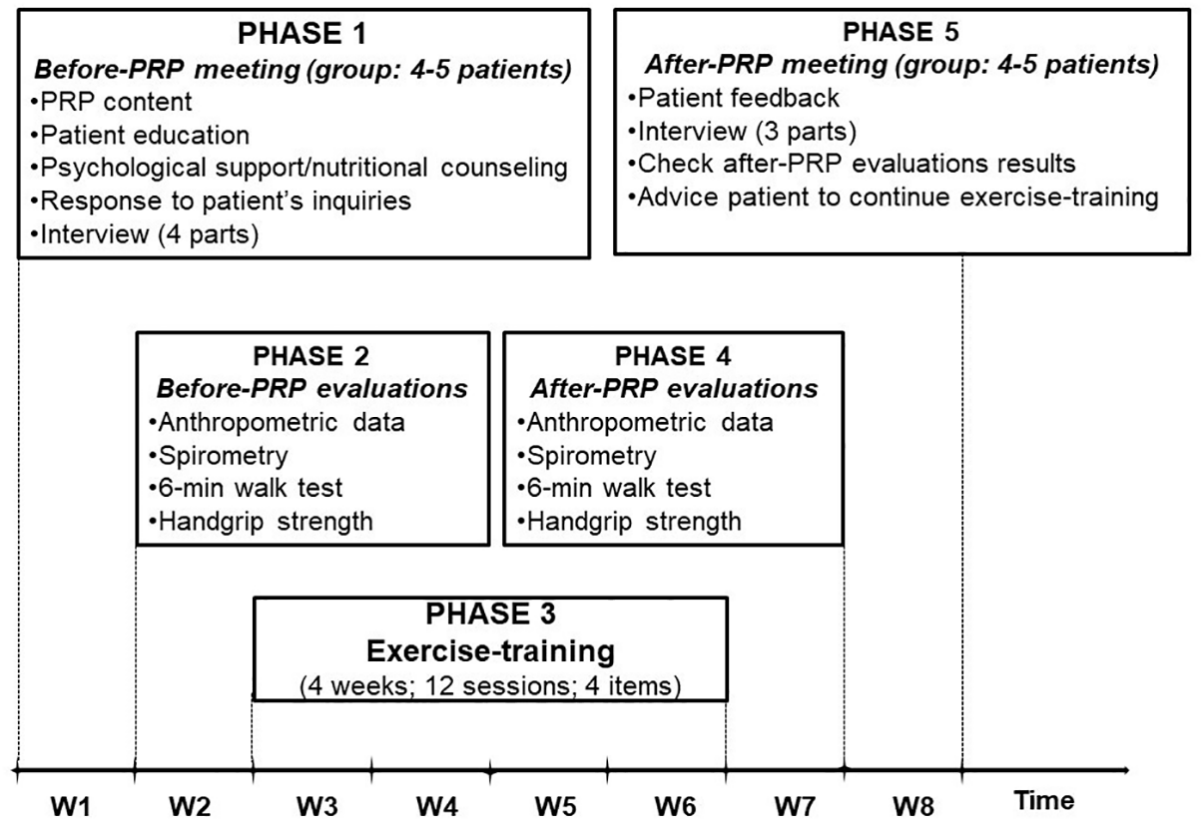
Five phases of the pulmonary rehabilitation program (PRP).

The PRP was ‘inspired’ from previous international recommendations for COVID-19 PRPs, and for the practice of physical activity in chronically ill patients aged >50 years. ^25^^,^^42^^–^^45^ Once four to five consecutive patients agreed to participate in the PRP, they formed one group and began the PRP. Figure 2 summarizes the five phases of the study.

The first phase of the study ( Figure 2) was a meeting between two physicians ( *ET* and *WB* in the authors’ list) and a group of four to five patients. During this phase, the physicians explained the PRP content and its progress; and when applicable, they educated patients about how to manage some comorbidities such as diabetes mellitus and/or arterial hypertension, and encouraged smokers to stop smoking. The physicians also related psychological support, such as how to manage emotional distress and/or post-traumatic stress disorder, and how to cope with COVID-19. ^46^ Moreover, the physicians also presented some nutritional advice ^47^^,^^48^ and responded to patients’ inquiries. During the end of this phase, each patient was interviewed separately. The interview, which included four parts [detailed in the appendices A (French/Arabic version) and B (translated English version)], was prepared in local Arabic dialect, and questions were asked by the same trained physicians ( *ET* and *WB* in the authors’ list). For each patient, the questions were repeated by the same interviewer bPRP and aPRP. The duration of the interview was approximately 30 minutes for each patient. The first part of the interview was derived from the American thoracic society questionnaire ^49^ and was performed only bPRP. This first part involved clinical (schooling and socioeconomic levels, lifestyle habits, medical and surgical history) and COVID-19 (date of RT-PCR, hospitalization, number of days bPRP, imaging) data. Two schooling-levels were arbitrarily defined **:** low (illiterate and primary school) or high (secondary and university). The socioeconomic level was determined according to the patient’s profession, and two levels were defined: unfavorable or favorable. Cigarette smoking was evaluated in pack-years, and patients were classified into two groups: non-smoker (<five pack-years), and smoker (≥five pack-years). Hospital stay was the number of days of hospitalization for COVID-19 management. The number of days bPRP represented the number of days between COVID-19 diagnosis (day of RT-PCR) and the first day of the onset of exercise training. The second part of the interview concerned the evaluation of the HRQoL via the VQ11. ^50^ VQ11 is a valid French questionnaire providing a reliable measure of HRQoL, which has been validated in patients with chronic respiratory conditions. ^50^ The VQ11 includes 11 items divided into three components (functional=three questions; psychological=four questions; and relational=four questions), and its score ranges from 11 to 55. ^50^ The VQ11 Arabic version was used. ^51^ The third part of the interview aimed to determine the current presence of and tendency towards anxiety or depression at the time of evaluation via the hospital anxiety and depression (HAD) questionnaire. ^52^ The latter includes 14 questions (score range 0–3); scored to separately estimate the anxiety and depressive status (seven questions each). The individual score for anxiety and depression subscales may vary from 0 to 21. The HAD Arabic version was used. ^53^ A HAD anxiety or depression score ranging between 0 and 7 was considered to be normal, a score between 8 and 10 indicated borderline-abnormal (borderline case) anxiety or depression, and a score of 11 or more indicated abnormal (case) anxiety or depression. ^54^ The fourth part of the interview concerned the Voorrips questionnaire, ^55^ which objectively evaluated the level of physical activity. This questionnaire is reproducible, and its score is positively correlated with the 24-hour measurement of the physical activity quantified by the use of a pedometer. ^55^ The Voorrips questionnaire includes 51 questions divided into three parts, evaluating the respondents’ daily sports and leisure physical activities. When added, the three parts give the total physical-activity score. ^55^ The Arabic version of the Voorrips questionnaire is not yet validated, but it was used in previous Tunisian studies. ^56^^,^^57^

The second phase of the study ( Figure 2) was reserved to perform evaluations/tests to gather anthropometric data including age, height (cm), weight (kg), and body mass index (BMI, kg/m ^2^), as well as spirometry, a 6-minute walk test, and handgrip strength. The data of the last three tests were explored in the first part of the project. ^24^ The obesity status was noted, in which underweight is BMI<18.5 kg/m ^2^, normal weight is BMI=18.5–24.9 kg/m ^2^, overweight is BMI=25.0–29.9 kg/m ^2^, and obesity is BMI≥30.0 kg/m ^2^. ^58^

**Figure 3.**
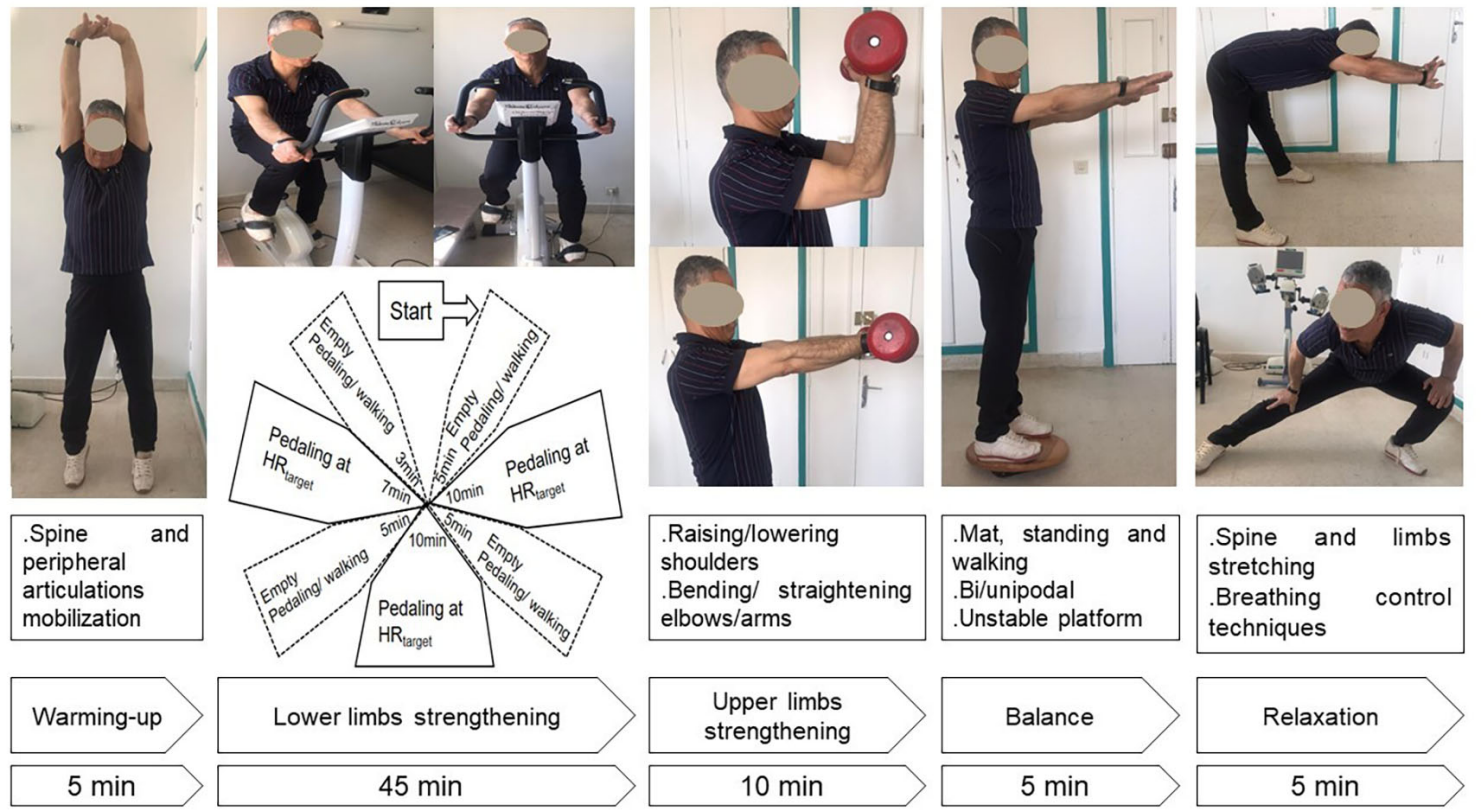
Description of an exercise training session. HR: heart rate.

The third phase of the study ( Figure 2) was reserved for the exercise training, which consisted of three sessions per week for four weeks (12 sessions of 70 minutes each). Exercise training was performed in four groups of four or five patients. A typical exercise training session included the following five items ( Figure 3):

i) *Item 1* involved warming-up for five minutes, including light exercises such as walking slowly, mobilization of cervical, lumbar spine, and peripheral joints.
ii) *Item 2* was comprised of lower limbs strengthening for 45 minutes such as aerobic training on an ergocycle. The cycling intensity was standardized and personalized using a heart rate (HR) monitor. The HR target was the HR determined at the end of the 6-minute walk test (±5 bpm). The HR monitor alarms were set around the HR target. The patients were asked to gradually reach their HR targets during the first five minutes and to maintain pedaling for 10 minutes at this intensity. Then, they were asked to return to empty pedaling or walking at their own pace for five minutes. They were again asked to complete one cycle of 10 minutes of target HR training and five minutes of active recovery (e.g., empty pedaling or walking at their own pace). To complete this item, the last cycle involved seven minutes of target HR training and three minutes of active recovery.
iii) *Item 3* was upper limbs strengthening for 10 minutes. Various muscle groups of the upper limbs performed sets of ten repetitions (e.g., raising and lowering shoulders, shoulder blade stabilization, bending and straightening elbows, raising arms). These exercises were performed without load during the first exercise training sessions, then with dumbbells of increasing weights along exercise training ( https://www.respir-sud.com/english/rehabilitation/12/105/2/D%c3%a9monstration.html).
iv) *Item 4* consisted of balance posture and proprioception exercises for five minutes. Several exercises were performed to improve balance posture, proprioception, coordination, and stability. Positions exercises included floor exercises, seated, and standing exercises and were varied between sessions. Exercises of increasing difficulty were performed along the exercise training, such as static and dynamic standing and walking exercises on a mat, as well as bipodal and unipodal exercises on an unstable platform ( https://www.respir-sud.com/english/rehabilitation/12/105/2/D%c3%a9monstration.html).
v) *Item 5* was a five-minute relaxation session. Numerous exercises involving spine and limbs stretching (e.g., standing stretch, cat back exercises, sphinx position) and breathing exercises (e.g., controlled diaphragmatic breathing, coordination between inspiratory and expiratory times) were performed ( https://www.respir-sud.com/english/rehabilitation/12/105/2/D%c3%a9monstration.html).

During each exercise training session, therapeutic education was carried out to strengthen the patients’ adherence to the lifestyle counseling provided during the bPRP meeting, for example management of comorbidities, encouraging smoking cessation when applicable, psychological support, and nutritional counseling. ^47^ All exercise training items were performed on patients not wearing a facemask.

The fourth phase ( Figure 2) was reserved to evaluations/tests similar to the second phase. During the fifth phase ( Figure 2), the following issues were tackled: patients’ feedback, questionnaires (as conducted during the first step, except the first part of the interview), checking the results of aPRP evaluations, and advising patients to continue exercise training.

### Sample size and statistical analysis

The sample size was calculated according to this formulae ^59^:

$$N=\frac{\left(Z_{\alpha }p\left(1-p\right)\right)}{i^{2}}$$

where ‘ *N*’ is the required number of patients, ‘ *Z _α_*’ is the normal deviates for a type I error (equal to 1.64 for 95% confidence level), ‘ *p*’ is the percentage of improvement of the main outcome (HRQoL score) aPRP in COVID-19 patients, and ‘ *i’* is the precision ( *i*=0.15). According to a French study, ^27^ the VQ11 total score of trained COVID-19 patients (N=39, mean age: 48 years) improved by 13.8%, from 29±10 bPRP to 25±10 aPRP (p=0.138). The application of the aforementioned data in the formula gave a number of 14 patients. We assumed that 20% of patients may not attend the exercise training sessions or the aPRP evaluation session, and therefore the revised sample was 18 patients (14/(1-0.20)).

Quantitative and categorical data were presented as mean±standard deviation (SD) 95% confidence interval and percentage, respectively. For each quantitative data including VQ11 scores (functional dimension, psychological dimension, relational dimension, total score), HAD anxiety and depression scores, Voorrips questionnaire (daily activity, physical activity, leisure activity, total score), a ΔPRP was calculated. The Wilcoxon matched pairs test and the one-sided chi squared test were used to compare the quantitative and categorical data bPRP and aPRP, respectively. PRP was considered ‘efficient’ if the ΔPRP change of the VQ11 total score (i.e., main outcome) is statistically significant. All statistical procedures were performed using statistical software (StatSoft, Inc. (2014). STATISTICA (data analysis software system), version 12. www.statsoft.com, 011after- PRPbefore-RRID: SCR_014213). The significance level was set at p<0.05.

## Results

Among the 68 COVID-19 patients discharged from COVID-19 hospital units, 18 volunteered to participate in the study. Among them, 14 patients completed the full PRP ( Figure 1).

The mean±SD (95% confidence interval) of age, height, weight, and BMI were 61±4 (59 to 64) years, 1.70±0.04 (1.67 to 1.72) m, 89±16 (80 to 99) kg, and 31.0±5.2 (28.0 to 34.0) kg/m ^2^, respectively. The frequencies of patients with normal weight, overweight, and obesity, were 7%, 50%, and 43%, respectively. The schooling and socioeconomic levels were low and unfavorable in 40% and 43% of patients, respectively, and 64% of patients were smokers (mean±SD of pack-years: 24±18 (8 to 39)). The frequencies of patients with diabetes mellitus, arterial hypertension, chronic obstructive pulmonary disease, dyslipidemia, and dysthyroidism, were 57%, 43%, 36%, 21%, and 7%, respectively. Table 1 details the COVID-19 data and severity classification of the 14 patients.

**Table 1.** Coronavirus disease 2019 (COVID-19) data and severity classification of the 14 patients.

Data		Unit/category	Values
Number of days before the pulmonary rehabilitation program		Days	83±30 (65 to 100)
Hospital stay		Days	17±7 (10 to 17)
Classification severity of COVID-19	Chest computed tomography	Extensive	29
		Severe	71
	Clinical	Moderate	21
		Severe	79

Quantitative and categorical data were mean±standard deviation (95% confidence interval) and percentage, respectively.

Table 2 details the impact of PRP on HRQoL, HAD anxiety and depression scores, and physical activity scores. It concluded that the means of the functional, psychological, and relational dimensions, and the VQ11 total score decreased significantly by 1.79, 2.00, 1.57, and 5.36 points, respectively. Additionally, the means of the HAD anxiety and depression scores decreased significantly by 2.07 and 2.57 points and the percentage of COVID-19 patients with abnormal HAD anxiety decreased significantly from 21% to 0%. Furthermore, the means of the physical activity and total scores increased significantly by 1.75 and 1.78 points, respectively.

**Table 2.** Impact of pulmonary rehabilitation program (PRP) on health-related quality of life, hospital anxiety depression, and physical activity scores (N=14 patients with coronavirus disease 2019).

Scores/data	Unit/levels	Before-PRP	After-PRP	ΔPRP	p value
**Health-related quality of life (VQ11 questionnaire)**					
Functional dimension	Absolute value	7.93±3.83 (5.72 to 10.14)	6.14±2.54 (4.68 to 7.61)	–1.79±1.58 (–2.70 to –0.87)	0.0033 ^*^
Psychological dimension	Absolute value	10.29±4.05 (7.95 to 12.62)	8.29±2.49 (6.85 to 9.73)	–2.00±2.15 (–3.24 to –0.76)	0.0108 ^*^
Relational dimension	Absolute value	7.71±3.69 (5.58 to 9.84)	6.14±2.51 (4.70 to 7.59)	–1.57±1.50 (–2.44 to –0.70)	0.0077 ^*^
Total score	Absolute value	25.93±10.59 (19.81 to 32.05)	20.57±6.89 (16.59 to 24.55)	–5.36±3.97 (–7.65 to –3.06)	0.0015 ^*^
**Hospital anxiety depression**					
Anxiety	Absolute value	6.71±4.48 (4.13 to 9.30)	4.64±3.23 (2.78 to 6.50)	–2.07±2.40 (–3.46 to –0.69)	0.0076 ^*^
Depression	Absolute value	7.00±5.16 (4.02 to 9.98)	4.43±3.32 (2.51 to 6.35)	–2.57±3.08 (–4.35 to –0.79)	0.0058 ^*^
Anxiety levels	Normal	50	79	-	0.0544
	Borderline case	29	21	-	0.3125
	Abnormal case	21	0	-	0.0350 ^*^
Depression levels	Normal	64	79	-	0.1897
	Borderline case	21	14	-	0.3130
	Abnormal case	14	7	-	0.2729
**Physical activity scores (Voorrips questionnaire)**					
Daily activity	Absolute value	1.50±0.48 (1.23 to 1.77)	1.54±0.49 (1.26 to 1.83)	0.04±0.19 (–0.07 to 0.15)	0.3268
Physical activity	Absolute value	1.83±3.13 (0.02 to 3.64)	3.58±3.30 (1.68 to5.49)	1.75±2.44 (0.34 to 3.16)	0.0251 ^*^
Leisure activity	Absolute value	0.96±1.15 (0.30 to 1.63)	0.95±1.15 (0.28 to1.61)	–0.02±0.40 (–0.25 to 0.21)	0.6547
Total score	Absolute value	4.29±3.23 (2.43 to 6.16)	6.07±3.85 (3.85 to 8.29)	1.78±2.65 (0.25 to 3.30)	0.0341 ^*^

ΔPRP: after-PRP value minus before-PRP value. Quantitative and categorical data were mean ± standard deviation (95% confidence interval), and percentage, respectively.

* p value<0.05 (Wilcoxon matched pairs test or one-sided chi squared test): before-PRP vs. after-PRP.

## Discussion

The present North African study demonstrated that a PRP improved HRQoL, HAD, and the physical activity of Tunisian COVID-19 patients. To the best of the authors’ knowledge, this is the first African and Arab study investigating the impact of a PRP on post-COVID-19 patients in terms of social disadvantages and physical activity. The methodology and main outcomes of some similar studies including a single group of COVID-19 patients, and case-control studies are detailed in Table 3 and Table 4, respectively.

**Table 3.** Methodology and main outcomes of some studies including a single group of COVID-19 patients and evaluating the impacts of pulmonary rehabilitation program (PRP) on social disadvantage data.

1 ^st^ author (Yr), [country], (reference)	a. Study design (Type PRP) b. Participants, N (male) c. Age (Yr)	Outcomes	Summary of findings
**Betschart (2021)** **[Switzerland]** ^26^	**a.** Pilot study (outpatient) **b.** 12 (8) **c.** 61 (26-84) ^**a**^	**PA/FS**: PCFS **HRQoL**: EQ-5D-5L VAS	Improve in PCFS ^*^ Improve in EQ-5D-5L VAS ^*^
**Gloeckl (2021)** **[Germany]** ^29^	**a.** Prospective observational cohort study (rehabilitation unit) **b.** 50 (22): mild/moderate: 24 (4), severe/critical: 26 (18) **c.** Mild/moderate: 52 (47-56) ^**c**^, severe/critical: 66 (60-71) ^**c**^	**PS**: GAD-7, PHQ-9 **HRQoL**: SF-36	**Mild/moderate**: no improve **Severe/critical**: improve in GAD-7 ^**β**^, PHQ-9 ^**β**^ and SF-36 mental component ^**β**^ ^**,**^ ^¥^
**Daynes (2021)** **[United Kingdom]** ^28^	**a.** Observational study (outpatient) **b.** 30 (16) **c.** 58 **±**16 ^b^	**PA/FS**: FACIT (fatigue) **PS:** HAD **HRQoL**: EQ 5D	Improve in FACIT ^*^
**Puchner (2021)** **[Austria]** ^32^	**a.** Observational multicenter study (rehabilitation unit) **b.** 23 (16) **c.** 57 **±**10 ^b^	**PA/FS**: BI **PS**: HAD, IES	Improve in BI *
**Bouteleux (2021)** **[France]** ^27^	**a.** Observational longitudinal study (outpatient) **b.** 39 (17), PFS: 29 (11), NPFS: 10 (6) **c.** 48 **±**15 ^b^	**PS**: HAD **HRQoL**: VQ11	Improve in VQ11 ^*^
**Piquet (2021)** **[France]** ^31^	**a.** Retrospective study (rehabilitation unit) **b.** 100 (66) **c.** 66±22 ^**c**^	**PA/FS**: BI	Improve in BI ^*^

BI: barthel index dyspnea. COVID-19: coronavirus disease 2019. EQ 5D: EuroQual 5 domains. EQ-5D-5L: Euroqol 5 domains. FACIT: functional assessment of chronic illness therapy. FS: functional state. GAD-7: generalized anxiety disorder-7 questionnaire. HAD: hospital anxiety and depression scale. HRQoL: health-related quality of life. IES: impact of event scale. N: number. PA: physical activity. PCFS: post-COVID-19 functional status scale. PHQ-9: patient health questionnaire 9. PS: psychological state. SF-36: short form 36. VAS: visual analogue scale. VQ11: health quality of life questionnaire. Yr: year.

Data were:

^a^ Median (minimum-maximum);

^b^ Mean ±SD;

^c^ Median (interquartile range).

* p<0.05: before PRP vs. after PRP. For the study of Gloeckl *et al*.:

^**α**^ p<0.05 before PRP vs. after PRP for the group mild/moderate;

^**β**^ p<0.05 before PRP vs. after PRP for the group severe/critical;

^**¥**^ p<0.05 between-group difference mild/moderate vs. severe/critical for the same period.

**Table 4.** Methodology and main outcomes of some studies case-control studies aiming to evaluate the impacts of pulmonary rehabilitation program (PRP) on social disadvantage data of COVID-19 patients.

1 ^st^ author (Yr) [country](reference)	a. Study design (Type PRP) b. Participants, N (male) c. Age (Yr)	Outcomes	Summary of findings: comparison
**Liu (2020)** **[China]** ^33^	**a.** Randomized controlled trial (outpatient) **b.** Intervention: 36 (24), Control: 36 (25) **c.** Intervention: 69±8 ^a^, Control: 69±8 ^a^	**PA/FS:** FIM **PS:** SAS, SDS **HRQoL:** SF-36	**Controls:** no improve **Intervention:** improve in SAS ^**α**^ ^**¥**^ and SF-36 ^**α**^ ^**¥**^
**Spielmanns (2021)** **[Switzerland]** ^34^	**a.** Interventional study (rehabilitation unit) **b. PG**: 99 (57), **LG**:419 (206) **c. PG**: 68±10 ^a^, **LG**: 69±11 ^a^	**PA/FS:** CIRS and FIM **PS:** HAD **HRQoL:** CRQ, FT	**PG:** improve in FIM ^**Δ**^ ^**£**^ and.FT ^**Δ**^ ^**£**^ **LG:** improve in FIM ^**δ**^ **and** FT ^**δ**^

CIRS: cumulative illness rating scale. COVID-19: coronavirus disease 2019. CRQ: chronic respiratory questionnaire. FIM: functional independence measure. FS: functional state. FT: feeling thermometer. HAD: hospital anxiety and depression scale. HRQoL: health-related quality of life. LG: lung diseases group. N: number. PA: physical activity. PG: post-COVID-19 group. PS: psychological state. SAS: self-rating anxiety scale. SDS: self-rating depression scale. SF-36: short-form 36. Yr: year.

Data were:

^a^ Mean±SD;

^*^ p<0.05; For the study of Liu *et al*.:

^**α**^ p<0.05 before-PRP vs. after-PRP for the same group cases;

^**¥**^ p<0.05 between-group difference cases vs. controls for the same period. For the study of Spielmanns *et al*.:

^**Δ**^ p<0.05 before-PRP vs. after-PRP for the PG group;

^**δ**^ p<0.05 before-PRP vs. after-PRP for the LG group;

^**£**^ p<0.05 between-group difference PG vs. LG for the same period.

### Impact of PRP on HRQoL

The three dimensions (functional, psychological, and relational) and the total score of the VQ11 increased after the PRP, by 1.79, 2, 1.57, and 5.36 points, respectively ( Table 2). These results are similar to those reported by Bouteleux *et al*. ^27^ In the latter study, the mean±SD of the VQ11 total score and the functional dimension score were improved by four points, from 29±10 bPRP to 25±10 aPRP (p=0.041), and two points, from 10±3 bPRP to 8±3 aPRP (p<0.001) in mild COVID-19 outpatients. However, Bouteleux *et al*. ^27^ reported no statistical changes in the psychological (from 11±4 bPRP to 10±4 aPRP (p=0.264)) and relational dimensions (from 9±4 bPRP to 8±4 aPRP (p=0.162)). Across similar studies, several assessment tools of HRQoL were used, including the Euroqol five domains (EQ 5D), EQ 5D five levels (EQ-5D-5L) visual analogue scale (VAS), ^26^^,^^28^ or SF-36 scale. ^29^^,^^33^ Betschart *et al*. ^26^ reported a statistically significant improvement in HRQoL from a mean of 65% (bPRP) to 81% (aPRP) on the EQ-5D-5L VAS (0–100%, 100% representing “the best health you can imagine”). Gloeckl *et al.* ^29^ noted that HRQoL was improved significantly only in patients with severe/critical COVID-19 (compared to mild/moderate COVID-19) in the mental component sum score of the SF-36 (from 38.5 bPRP to 52.9 aPRP points). Daynes *et al*. ^28^ highlighted that the EQ 5D thermometer improved by 8±19 (p=0.05) after six weeks of a PRP using exercise and education. Liu *et al*. ^33^ concluded that the eight dimensions of the SF-36 scores in elderly COVID-19 patients were statistically significant within the intervention group that underwent six weeks of a PRP, between the intervention and control groups.

From a public health viewpoint, it is central to determine the level of HRQoL in order to suggest a theoretical base for the expansion of appropriate policies and courses to increase well-being and avoid the frequent difficulties related to HRQoL. ^60^ HRQoL is an increasingly important health goal, and it is an independent predictor of health outcome. ^61^ The effectiveness of a PRP in increasing physical performance and HRQoL could be explained by the improvement in dyspnea, lung function, or the 6-minute walk distance. ^62^ Bardakci *et al.* ^62^ noted that physical health parameters of SF-36 are lower in patients with a low 6-minute walk distance and low spirometry data. At the post-recovery stage, most COVID-19 patients retain an altered HRQoL, which poses challenges to patients, and health professionals, ^12^ thus having a major impact on economic trends in almost all sectors. ^63^ Predictors for COVID-19-induced deficits in HRQoL after hospitalization are yet to be determined in research. ^26^

### Impact of PRP on HAD anxiety and depression

As done in some related studies ^27^^,^^28^^,^^32^^,^^34^ ( Table 3 and Table 4), anxiety and depression were evaluated using the HAD questionnaire. ^54^ The means of the HAD anxiety and depression scores decreased significantly by 2.07, and 2.57 points, respectively, and the percentage of COVID-19 patients with abnormal HAD anxiety decreased significantly from 21% to 0%. These findings are different to these reported in some studies, ^27^^,^^28^^,^^32^ where no improvement was noted in both HAD anxiety and depression scores ( Table 3 and Table 4). First, in the study of Daynes *et al.,* ^28^ the increases in the HAD anxiety and depression mean±SD scores by 0±4 (p=0.5) and 1±4 (p=0.1) were not statistically significant. Second, Bouteleux *et al.* ^27^ reported that the increases in the mean±SD scores of HAD anxiety (from 8±3 bPRP to 8±0 aPRP, p=0.941) and depression (from 6±4 bPRP to 5±0 aPRP, p=0.306) were not statistically significant. Third, Puchner *et al*. ^32^ noted that symptoms of depression and anxiety were not increased after multi-disciplinary inpatient PRP in 23 patients discharged after severe to critical COVID-19 infection. Other studies have used different scales, such as self-rating anxiety and depression scale (SAS, SDS, respectively) ^64^ and the generalized anxiety disorder-7 questionnaire (GAD-7), ^65^ and reported improvement only in anxiety. ^29^^,^^33^ For instance, Gloeckl *et al.,* ^29^ reported a slight but significant increase in the GAD-7 score by a median (interquartile range) of 1 (0–2, p=0.021) after three weeks of inpatient PRP in COVID-19 patients. In the study of Liu *et al.,* ^33^ SAS and SDS scores decreased after six weeks of a PRP in the intervention group, but only anxiety was statistically significant within and between groups (intervention vs. control), and SDS scores were not statistically significant within or between groups.

In our study, the improvement in anxiety and depression status could be related to the psychological support provided during the PRP (e.g., management of emotional distress, post-traumatic stress disorder, and strategies for coping with COVID-19). Moreover, anxiety and depression have been linked to reduced HRQoL. ^66^

### Impact of PRP on physical activity

The present study is the first to evaluate the impacts of a PRP on the physical activity of COVID-19 patients evaluated using a generic questionnaire ^55^ ( Table 2). In the literature, some related studies have evaluated the impacts of PRPs on functional impairment using some functional assessment scales including general scales (e.g., functional independence measure, ^33^ functional assessment of chronic illness therapy) ^28^ or specific scales (e.g., post-COVID-19 functional status scale ^26^ ( Table 3 and Table 4)), and have reported divergent conclusions. Indeed, Liu *et al*. ^33^ did not note any improvement in the activities of daily living assessed by the functional independence measure scale. ^33^ However, some authors reported an improvement in the functional status. ^26^^,^^28^ In fact, symptoms such as dyspnea in chronic respiratory diseases could cause reductions in the activities of daily living, thus leading to dependence and disability. ^67^ By improving these symptoms, a PRP in COVID-19 patients could restore their ability to take care of themselves. ^4^^,^^15^^,^^68^ Physical activity continues to be the best method to preserve well-being, guarantee daily activities, and preserve good function of the cardiorespiratory and muscular chain. ^45^ From a public health awareness perspective, it is vital to reconnoiter the level of physical activity in the interest of providing a theoretical foundation for the expansion of appropriate guidelines to ameliorate health and turn aside the numerous problems related to physical inactivity. ^60^

The inconsistencies noted between our findings and these of some similar studies ( Table 3 and Table 4) could be clarified by some points related to dissimilarities in:

i) Study designs, including case control ^33^ vs. prospective observational cohort ^29^ studies.
ii) PRP locations, such as inpatient ^32^ or outpatient ^26^ PRP.
iii) Inclusion criteria, for example inclusion of COVID-19 patients of different ages e.g., elderly (≥ 65 years) ^33^ or middle-aged (48±15 years) ^27^], or both males and females. ^27^^,^^28^
iv) COVID-19 patients’ profiles, including their comorbidities or lack of them and/or the disease severity stages, from mild/moderate to severe/critical. ^29^
v) PRP components, such as exercise training alone ^27^ vs. exercise training and education. ^26^^,^^30^
vi) Durations and/or frequencies of PRP, for example two sessions per week and 16 sessions ^26^ vs. five sessions per week and three sessions. ^29^
vii) Time periods between the diagnosis of COVID-19 and the beginning of PRP (e.g., late PRP for long-COVID-19 ^28^ vs. early PRP for acute COVID-19 ^32^).

### Strengths and limitations

The present study has two strong points and three main limitations. The first strong point was the fact that the study was led in an outpatient unit in a low-income country and the different components of a PRP were performed, including exercise training, education, and nutritional counseling. The second strong point was the calculation of the sample size according to a predictive formula. ^59^ The first major limitation concerns the lack of a randomized COVID-19 control-group due to ethical considerations during the COVID-19 pandemic. Indeed, while few similar studies included a control group ^33^^,^^34^ ( Table 4), several other studies ^26^^–^^32^ included only one group ( Table 3). The lack of a control-group did not allow us to ‘affirm’ that our findings are solely attributable to the PRP. For that reason, we recognize that the positive impacts of the PRP could be interpreted as a natural remission of COVID-19. Nevertheless, the initial symptoms in our patients presented almost three months before the PRP ( Table 1). Consequently, we relate these improvements principally to the impact of the PRP, which also comprised specific interventions focusing on disease management and coping with COVID-19 and its sequelae. ^29^ The second limitation concerns the use of the VQ11 questionnaire to evaluate the HRQoL. ^27^ The VQ11 is mainly validated in COPD patients and is not proposed for being used in the general population. ^50^ However, the VQ11 is usually used in our departments, ^51^ easily administered, and seems reliable with the indication of a PRP in our study as it precisely assesses the alteration of HRQoL related to respiratory disability. ^27^ The third limitation involves the use of a non-validated Arabic version of the Voorrips questionnaire. ^55^ However, the Arabic version of that questionnaire was previously used in some Tunisian studies. ^56^^,^^57^ Future research to address the aforementioned limitations is needed.

## Conclusion

Four weeks of a PRP in post-COVID-19 patients is efficient even in a low-income country, such as Tunisia. This Tunisian study demonstrated that a PRP performed in patients with post-COVID-19 is associated with marked improvements in HRQoL, HAD anxiety and depression, and physical activity scores. Pulmonary rehabilitation has imposed itself as a standard of care for the treatment of post-COVID-19 patients. Future randomized studies are needed to assess the effectiveness and long-term benefits of PRP in post-COVID-19 patients.

## Ethical approval

This study was approved by the ethics committee of Farhat HACHED Hospital (Approval number FH2502/2021).

## Informed consent

Written informed consent was obtained from all patients after receiving an explanation of the study.

## Data Availability

Zenodo: Impact of a pulmonary rehabilitation program on social disadvantage and physical activity data of postCOVID19 patients: A North-African pilot study, https://doi.org/10.5281/zenodo.7191266. ^69^ The project contains the following underlying data:
- [Data of 14 patients.xls] (Excel file including the numerical data of the 14 patients). - [ **Appendix A**: Copy of the French/Arabic questionnaire] (Applied questionnaire). - [ **Appendix B**: Copy of the translated English questionnaire] (Translated questionnaire). Zenodo: STROBE checklist for ‘[Impact of a pulmonary rehabilitation program on social disadvantage and physical activity data of postCOVID19 patients: A North-African pilot study], https://doi.org/10.5281/zenodo.7191266. ^69^ Data are available under the terms of the Creative Commons Attribution 4.0 International license (CC-BY 4.0).
