# Supplementary material for: Impact of a pulmonary rehabilitation program on social disadvantage and physical activity data of postCOVID19 patients: A North-African pilot study
Source: F1000Res. 2022 Oct 27;11:1226. [Version 1] doi: 10.12688/f1000research.126301.1 (PMC10403745; doi:10.12688/f1000research.126301.1)
Supplement: Peer review history

# Peer review: Reviewer response for version 1

**DOI:** 10.5256/f1000research.138698.r190403

**Published:** 2023-08-04

**Authors:** Mehdi Martani

**Affiliations:** 1 Department of physiology, Constantine Hospital, Constantine, Constantine Province, Algeria

I read with great interest this article on the role of rehabilitation in improving quality of life and physical activity in Tunisian patients with COVID19 .

 The problem addressed by the authors is of great importance, because the post COVID19 syndrome is a topical subject that remains until now poorly explored.

 Sections of the article are clear, consistent and balanced.

 The methodology of the study is very explicit with a detailed rehabilitation protocol. 

 However, I have only one comment regarding the use of the VQ11 questionnaire to assess the quality of life of patients with COVID19, knowing this questionnaire was originally developed for the assessment of the quality of life of patients with COPD. My my concern is to have explanations or justifications regarding the use of the VQ11 questionnaire for the assessment of the quality of life of patients with post COVID-19 syndrome.

Is the work clearly and accurately presented and does it cite the current literature?

Yes

If applicable, is the statistical analysis and its interpretation appropriate?

Yes

Are all the source data underlying the results available to ensure full reproducibility?

Yes

Is the study design appropriate and is the work technically sound?

Yes

Are the conclusions drawn adequately supported by the results?

Yes

Are sufficient details of methods and analysis provided to allow replication by others?

Yes

Reviewer Expertise:

Pulmonary function testing -  Respiratory physiology

I confirm that I have read this submission and believe that I have an appropriate level of expertise to confirm that it is of an acceptable scientific standard.

# Peer review: Reviewer response for version 1

**DOI:** 10.5256/f1000research.138698.r160406

**Published:** 2023-02-03

**Authors:** Yacine Ouahchi

**Affiliations:** 1 Service de pneumologie, CHU La Rabta, Tunis, Tunisia

This study reports the impact of a pulmonary rehabilitation program (PRP) on social disadvantage (HRQoL, anxiety and depression) and physical activity in Tunisian COVID-19 patients. The authors found that PRP improve HRQoL, HAD and physical activity in their studied population. The topic is interesting, because the results of this study add to our knowledge about the benefits of a PRP in COVID 19 patients. Moreover, this study is the first conducted in a low-income country, allowing doing comparisons with results of other studies in high-income countries.

 Overall, the manuscript is well written, with a perfect coherence between its different parts. I consider then that it may be indexed in the present form. Below are some comments:

 Introduction

 The authors described clearly the COVID and the post COVID syndrome. The problem was also briefly and clearly defined as well as the aims of the study.

 Methods

 The study population was clearly defined with justifying the sample size. The study protocol comprised 5 phases which were clearly described in all what concerned evaluations and the exercise training session.

 Results

 Results were reported and resumed in a table showing the changes induced by PRP in HRQoL, HAD and physical activity scores in the studied population.

 Discussion:

 The authors discussed the results and compared them to those of other studies. They also reported the strengths and limitations of the study.

Is the work clearly and accurately presented and does it cite the current literature?

Yes

If applicable, is the statistical analysis and its interpretation appropriate?

Yes

Are all the source data underlying the results available to ensure full reproducibility?

Yes

Is the study design appropriate and is the work technically sound?

Yes

Are the conclusions drawn adequately supported by the results?

Yes

Are sufficient details of methods and analysis provided to allow replication by others?

Yes

Reviewer Expertise:

Lung function testing and sleep medicine

I confirm that I have read this submission and believe that I have an appropriate level of expertise to confirm that it is of an acceptable scientific standard.

# Response

**Published:** 2023-02-03

**Authors:** Helmi BEN SAAD

**Affiliations:** Université de Sousse, Tunisia

All the authors are very happy to note that the reviewer appreciated our paper. No action is needed from our side. 

 Thanks
